# 655 Assessing Housing Status of Inpatient Admissions for Thermal Injuries: Insights from a Canadian Burn Unit

**DOI:** 10.1093/jbcr/iraf019.284

**Published:** 2025-04-01

**Authors:** Justin Lee, Izza Sattar, Shawn Dodd, Trent Schimmel, Sharada Manchikanti, Joshua Wong, Alexis Armour

**Affiliations:** University of Alberta; University of Alberta; University of Alberta; University of Alberta; Alberta Health Services; University of Alberta; University of Alberta

## Abstract

**Introduction:**

Houselessness has increased in urban regions since the Covid-19 pandemic, potentially posing a risk to the population in the northern climate. This study investigates how pre-injury living conditions influence health outcomes among thermally injured inpatients post-pandemic. We hypothesized that housing status at the time of injury and admission is a significant determinant of health in our patient population.

**Methods:**

A retrospective chart review was completed on patients admitted to the burn service at a level one trauma centre from January to December 2023. We examined demographics, injury types, and disposition among housed and unhoused inpatients. Primary outcome assessed was the total length of hospital stay (LOS). Secondary outcomes included frequency of discharges against medical advice (AMA). Statistical analysis was performed using t-test for continuous and chi-squared test for categorical variables.

**Results:**

Of 214 new admissions in 2023, there was a greater proportion of burn injuries at 196 (91.6%) compared to frost injuries at 18 (8.4%) (p = 0.001). The rate of thermal and frost burn requiring hospitalization for unhoused individuals was estimated at 2025 and 437 per 100,000 persons, respectively. Significantly greater proportion of housed vs unhoused patients experienced thermal burn injuries (145 vs 51, respectively). In contrast, the unhoused population experienced a greater number of frost injuries, versus the housed population (11 vs 7, p = 0.001). The unhoused population had a significantly greater frequency of leaving against medical advice (35.4%), compared to the housed (4.9%, P < 0.0001). When excluding the patients who left AMA from the analysis, the mean length of stay in the housed and unhoused burn service inpatients (17.2, and 21.7 days, respectively) was not significantly different (p = 0.15).

**Conclusions:**

In 2023, 26% of new thermal injury admissions were experiencing houselessness, demonstrating an increase compared to reported pre-pandemic levels. Our preliminary data demonstrate higher rates of unstable housing in frost injuries, compared with burn injuries, with a significant number of both groups leaving the hospital AMA. We plan to perform secondary outcome variable analyses to assess for contributing factors, while expanding our sample to include 2022 and 2024. In conclusion, housing status has a disproportionate effect on frostbite admissions in the northern climate, showing the significance of injury prevention with housing initiatives in Canada.

**Applicability of Research to Practice:**

Future analyses will include quality improvement projects to identify factors contributing to higher rates of AMA in unhoused patients, as well as cost-benefit analyses of healthcare costs vs temporary housing to inform Canadian policy decisions regarding this vulnerable population.

**Funding for the Study:**

N/A

